# Case report: Fat-containing liver metastases from retroperitoneal liposarcoma

**DOI:** 10.4103/0971-3026.41834

**Published:** 2008-08

**Authors:** Mahesh Prakash, Sameer Vyas, Alampady Krishna Prasad Shanbhogue, Mandeep Kang, Pranab Dey, Niranjan Khandelwal

**Affiliations:** Department of Radiodiagnosis and Imaging, Postgraduate Institute of Medical Education and Research, Chandigarh - 160 012, India; 1 Department of Cytology, Postgraduate Institute of Medical Education and Research, Chandigarh - 160 012, India

**Keywords:** Computed tomography, fine needle aspiration cytology, hepatic metastases, liposarcoma

## Case History

**Figure 1:**
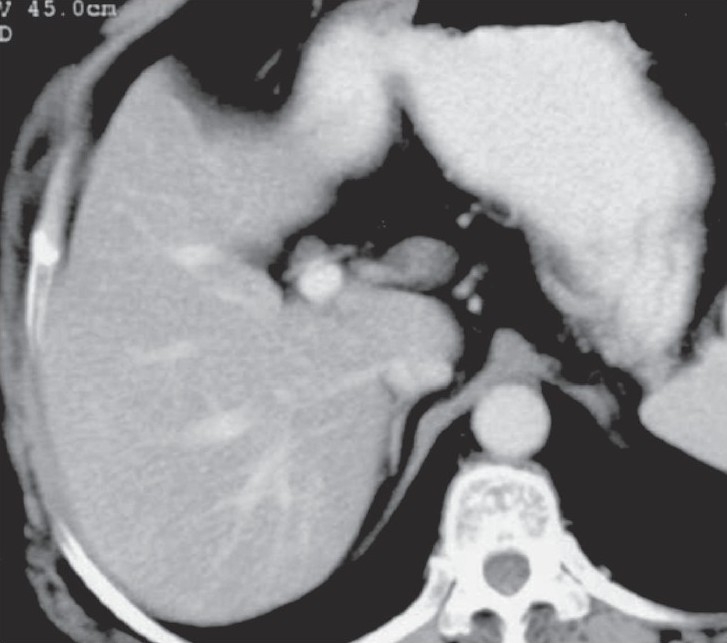
Axial contrast-enhanced CT scan of the liver done in May 2004 shows absence of focal hepatic lesions

A 54-year-old man, treated in the past for retroperitoneal liposarcoma, came for a follow-up CT scan. He had been operated upon for this condition in December 2000 and again, for a recurrence, in May 2004. On both occasions, imaging had shown a normal liver [Figure 1].

**Figure 2:**
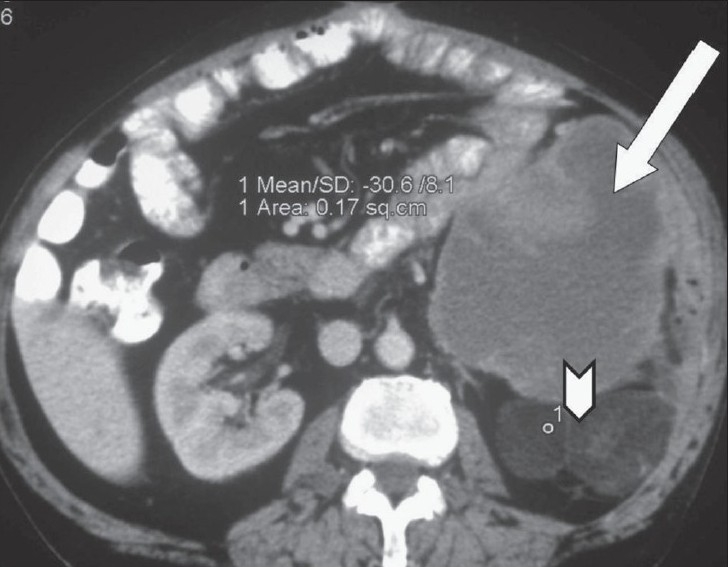
Axial contrast-enhanced CT scan shows a large, lobulated, heterogeneous mass in the left half of the retroperitoneum (arrow) with a fatty component posteriorly (arrowhead)

**Figure 3 (A, B):**
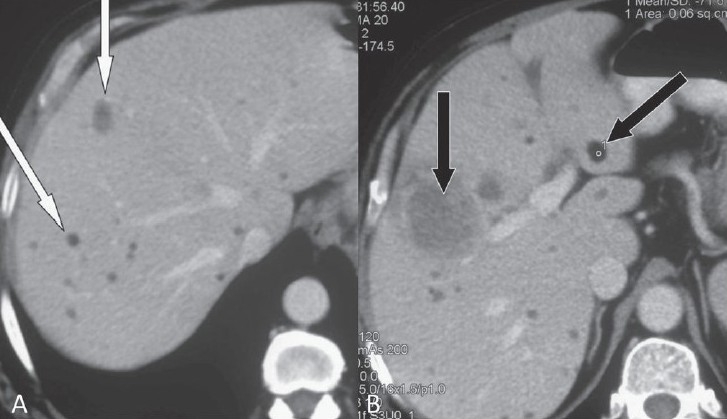
Axial contrast-enhanced CT scans show multiple, hypodense, focal lesions in both lobes of the liver with fatty attenuation (arrows)

A contrast-enhanced CT scan of the abdomen showed a large 14 × 12 cm mass in the retroperitoneum with heterogeneous attenuation [Figure 2]. This was suggestive of a recurrent liposarcoma. There were multiple focal hypodense lesions showing fat attenuation (−30 to −70 HU) in both lobes of the liver, without enhancement [Figure 3]. USG-guided fine needle aspiration cytology (FNAC) showed features consistent with metastatic sarcoma.

## Discussion

Liposarcoma is a malignant mesenchymal tumor which most often occurs in the fifth and sixth decades.[1] It commonly occurs in the retroperitoneum or the lower limb. Less frequently, it can develop in the upper limb or in the head and neck region.[2]

CT scan can suggest a diagnosis of liposarcoma when fat is detected within a retroperitoneal mass. Fat shows characteristic low attenuation (−10 or less HU) on CT scans. The amount of identifiable fat in liposarcoma varies widely.

The incidence of hepatic metastases depends on the histological subtype of the liposarcoma and the site of the primary tumor. Huang *et al.* found only one patient with liver metastases among 354 patients with retroperitoneal dedifferentiated liposarcoma. They have quoted the cumulative incidence of liver metastases to be between 1-18%.[3] In contrast, Sheah *et al.* have reported a much higher incidence of up to 33% in myxoid liposarcoma.[4] These tumors commonly present as single or multiple lobulated soft tissue masses, with or without macroscopic fat components.[4]

Finding fat in liposarcoma metastases is uncommon. A wide variety of hepatic mass lesions show fat attenuation on CT. These include benign conditions such as focal or geographic fatty change, pericaval fat, postoperative packing material (omentum), adenoma, focal nodular hyperplasia, lipoma, angiomyolipoma, and cystic teratoma as well as malignant liver lesions like hepatocellular carcinoma, primary and metastatic liposarcoma, and other metastases.[5] Identification of fat within a liver lesion may be crucial for characterizing some of these lesions. The pattern of fatty change (macroscopic *vs* intracellular lipid) and whether the lesion contains only fat (e.g., lipoma, postoperative packing material, focal steatosis) or fat and soft tissue (e.g., adenoma, angiomyolipoma, teratoma, primary and metastatic liposarcoma and hepatocellular carcinoma) are factors that are useful in narrowing down the differential diagnosis.[5] Although USG and CT scan can detect fat in the majority of the cases, MRI with chemical-shift imaging may be a better modality to detect fat, especially intracellular lipid.[6] Basaran *et al.*, in their review on fat-containing lesions of the liver, have described 16 different types of hepatic lesions that demonstrate fat within.[7]

In this case, local recurrence was found in both lobes of the liver, with multiple hypodense, fat-attenuation focal lesions on CT scan. This is an unusual finding, and the presence of fat was of help in pinpointing the nature of the metastases.
